# *In Utero* Chlordecone Exposure and Thyroid, Metabolic, and Sex-Steroid Hormones at the Age of Seven Years: A Study From the TIMOUN Mother-Child Cohort in Guadeloupe

**DOI:** 10.3389/fendo.2021.771641

**Published:** 2021-11-22

**Authors:** Gülen Ayhan, Florence Rouget, Frank Giton, Nathalie Costet, Léah Michineau, Christine Monfort, Jean-Pierre Thomé, Philippe Kadhel, Sylvaine Cordier, Alejandro Oliva, Luc Multigner

**Affiliations:** ^1^ Centre Hospitalier Universitaire (CHU) de Guadeloupe, Univ Antilles, Inserm, École des Hautes Études en Santé Publique (EHESP), Irset (Institut de recherche en santé, environnement et travail) -UMR_S 1085, Rennes, France; ^2^ Centre Hospitalier Universitaire (CHU) de Rennes, Univ Rennes, Inserm, École des Hautes Études en Santé Publique (EHESP), Irset (Institut de recherche en santé, environnement et travail) - UMR_S 1085, Rennes, France; ^3^ Assistance Publique - Hôpitaux de Paris (AP-HP) Hôpital Henri Mondor, Pôle Biologie-Pathologie, IMRB U955, Créteil, France; ^4^ Univ Rennes, Inserm, École des Hautes Études en Santé Publique (EHESP), Irset (Institut de recherche en santé, environnement et travail) -UMR_S 1085, Pointe à Pitre, France; ^5^ LEAE-CART (Laboratoire d’Ecologie Animale et d’Ecotoxicologie-Centre de Recherche Analytique et Technologique), Université de Liège, Liège, Belgium; ^6^ Center for Interdisciplinary Studies, National University of Rosario, Rosario, Argentina

**Keywords:** chlordecone, endocrine disruptors, *in utero* exposure, hormones, children

## Abstract

**Background:**

Chlordecone is an endocrine-disrupting chemical with well recognized estrogenic and progestagenic properties. This organochlorine insecticide was extensively used in the French West Indies from 1973 to 1993 to control the banana root borer. Due to its poor degradation in the environment, permanently polluted soil is responsible for the current contamination of the food chain and human beings. We aimed to examine the relationship of *in utero* exposure to chlordecone and thyroid (thyroid stimulating hormone [TSH], free tri-iodothyronine [FT3], free thyroxine [FT4]), metabolic (insulin growth-factor 1, leptin, adiponectin), and sex-steroid (dehydroepiandrosterone [DHEA], total testosterone [TT], dihydrotestosterone [DHT], estradiol [E2]) hormone levels in children at the age of seven years who participated in TIMOUN, an ongoing birth cohort in Guadeloupe.

**Methods:**

Chlordecone concentrations were measured in cord-blood at delivery. Thyroid, metabolic, and sex-steroid hormone levels were determined in the blood of children at seven years of age. Associations between *in utero* chlordecone exposure and hormone levels at seven years of age were assessed by multiple linear or logistic regression, controlling for confounding factors.

**Results:**

Among the study population (210 boys and 228 girls), chlordecone and hormone measurements were available for 124 boys and 161 girls. We found the third quartile of *in utero* chlordecone exposure relative to the lowest quartile to be associated with elevated TSH levels in girls and elevated DHEA, TT, and DHT levels in both sexes. Complementary non-linear analysis (spline regression) confirmed a significant non-linear trend for TSH in girls and DHEA and DHT in boys.

**Conclusion:**

*In utero* chlordecone exposure was associated with elevated levels of selected thyroid (TSH) and sex-steroid (DHEA, TT, and DHT) hormones at seven years in a non-monotonic dose response (inverted U) relationship. The implications for future health and reproductive function in puberty and adulthood should be determined.

## Introduction

Chlordecone is a persistent organochlorine insecticide that was extensively used in Guadeloupe and Martinique (French West Indies, FWI) from 1973 to 1993 to fight against the banana root borer (1). This pesticide undergoes no significant biotic or abiotic degradation in the environment (2). Although chlordecone has not been used since 1993, it persists in the soil of current and former banana fields where it has been spread. Simulation studies have shown that it would take up to 7 centuries for soils to be cleaned up through leaching (3). Chlordecone in soil is slowly drained by rainfall towards superficial water, ground water, and marine coastal waters and contaminates the terrestrial and aquatic ecosystems, including crops, livestock, and fishing products (4, 5). Most local animal and vegetable production and consumption is not influenced by the limited seasons that occur in the FWI, as in many tropical Caribbean areas. Therefore, human exposure to chlordecone in the FWI arises from the continuous consumption of contaminated foodstuffs. The analysis of blood samples has shown that a large proportion of the French West Indies population, both infants and adults, are contaminated by chlordecone (1, 6–8). Chlordecone crosses the human placental barrier, thus exposing the fetus during its development (9, 10).

Epidemiological studies conducted in the FWI have reported that chlordecone exposure is associated with an increased risk of prostate cancer in adult men, preterm birth in pregnant women, and altered growth, cognitive, motor or visual development in toddlers and children (7, 9–14).

Several biological mechanisms have been suggested to explain the unwanted effects of chlordecone on health. Chlordecone has the capacity to inhibit brain ATPases, as well as that to interact with multiple neurotransmitters (noradrenaline, dopamine, GABA, etc.) (15). Moreover, estrogenic and progestagenic-like properties have been clearly established both *in vivo* and *in vitro* (16–21). Chlordecone binds to estrogen receptors α (ERα) and β (ERβ), acting as an agonist of ERα and an antagonist of ERβ (22, 23). In addition to its interaction with nuclear ERs, chlordecone may activate alternative estrogen signaling pathways or other enzymes and receptors involved in steroid homeostasis (24–27). These data all support chlordecone an endocrine disruptor.

Endocrine-disrupting chemicals (EDCs) are exogenous agents that interfere with the synthesis, secretion, transport, metabolism, binding action, and/or elimination of natural blood hormones (28). Hormonal homeostasis is essential for a large variety of physiological processes, including growth, development, reproduction, energy balance, metabolism, and the regulation of body weight, among others (29). By interfering with hormonal homeostasis, EDCs may affect such physiological processes and lead to deleterious effects on many endocrine systems, with negative outcomes (30). Among the key characteristics of EDCs are those of being able to alter hormone synthesis, transport, metabolism, and clearance (31). Such alterations can result in changes in circulating hormone levels, which, in turn, can lead to negative health outcomes.

Few studies have investigated the relationship between chlordecone exposure and circulating hormone levels. No association was found among healthy adult Guadeloupian men between chlordecone exposure and circulating levels of dehydro-epiandrosterone (DHEA), DHEA-sulphate, androstenedione, androstenediol, total testosterone (TT), free and bioavailable testosterone, dihydrotestosterone (DHT), estrone, E1-sulphate, or estradiol (E2) (32). In the TIMOUN Mother-Child Cohort Study in Guadeloupe, *in utero* exposure to chlordecone has been shown to be associated with increased thyroid stimulating hormone (TSH) levels at three months of age, but only for boys, without modification of free tri-iodothyronine (FT3) or free thyroxine (FT4) levels (33).

The *in utero* period of development is a recognized temporal window of vulnerability during which EDCs may exert their effects, resulting in a large spectrum of disorders, some of which are sexually dimorphic, later in life (30). Here, we investigated the relationship between prenatal (*in utero*) exposure to chlordecone and the circulating levels of thyroidal (TSH, FT3, FT4), metabolic (Insulin growth-factor 1 [IGF-1], adiponectin, leptin), and sex-steroid (DHEA, TT, E2) hormones in children at seven years of age who participated in the follow-up of the TIMOUN Mother-Child cohort study.

## Materials and Methods

### Participants

This study was conducted in Guadeloupe (French West Indies), a Caribbean archipelago. The TIMOUN Mother-Child Cohort was established to investigate the consequences of prenatal (maternal or *in utero*) exposure to chlordecone on pregnancy and child development. From November 2004 to December 2007, 1,068 pregnant women from the general population were enrolled during their third-trimester prenatal visit at public health centers (University Hospital of Pointe-à-Pitre, General Hospital of Basse-Terre, and antenatal care units) (6). Questionnaires were administered at inclusion to assess their social, demographic, occupational, medical, and family characteristics, as well as lifestyle habits. At delivery, data concerning maternal diseases during pregnancy, delivery, and the health status and anthropometric characteristics of the newborn at birth were collected by the medical staff and maternal and cord blood samples were obtained. Follow-up visits of the children were conducted afterwards at various ages (3, 7, and 18 months) to evaluate the development of the children (9, 10, 33). When the children were seven years of age (May 2011 to October 2015), 1,033 mothers initially included in the cohort were contacted for a follow-up interview and a medical examination of their child. Among them, 589 (57% of the initial cohort) were interviewed and had their child examined at the University Hospital of Guadeloupe. During the medical examination of the children, face-to-face interviews were conducted with their mothers to collect information about the socio-economic context in which the child was growing and the health of the child, including medication intake. A blood sample was also collected from the children at the end of the examination. We excluded five children from the present study because of major congenital anomalies or severe diseases that could affect hormone levels. We then retained those for whom at least one blood hormone concentration was successfully measured at seven years of age, leaving 438 children (210 boys and 228 girls). Among them, a cord-blood chlordecone measurement was available for 285 (124 boys and 161 girls). No children were treated with hormonal or anti-hormonal medications.

The study was approved by the relevant ethical committee for studies involving human subjects (Comité de Protection des Personnes Sud-Ouest et Outremer III (n° 2011-AOOSSI-40). Each parent provided written informed consent.

### Blood Collection

Blood samples at birth (umbilical cord blood) and at seven years of age were collected in EDTA tubes and, after centrifugation, plasma samples were conserved in polypropylene Nunc^®^ tubes at -30°C. They were transferred on dry ice by airmail to the Center for Analytical Research and Technology (CART) at Liège University in Belgium for organochlorine analysis and to the steroid mass spectrometry platform of the Mondor Institute for Biomedical Research, Créteil, France, for hormone analysis.

### Organochlorine Analyses

Blood samples were analyzed for chlordecone, p,p´−dichlorodiphenyldichloroethylene (DDE, the major and most persistent metabolite of dichlorodiphenyltrichloroethane, DDT), and the non-dioxin-like polychlorinated biphenyl congener 153 (PCB-153) by high-resolution gas chromatography with Ni63 electron capture detection. Detailed information about the sampling, analysis, and quality assurance and control have been provided elsewhere (11, 34). Among PCBs, we selected PCB-153 because it correlates very well with the total PCB concentration in plasma (35). The analytical limit of detection (LD) was 0.06 μg/L for chlordecone in cord blood, 0.05 µg/L for DDE and PCB-153 in cord blood, and 0.02 μg/L for chlordecone in the children’s blood.

### Hormone Analyses

TSH, FT3, and FT4 were measured by immuno-radiometric assay (IM 3712 TSH Irma Kit, IM 1579 FT3, IM 1363 FT4, Beckman Coulter). Results were expressed as concentrations (milli-international units per liter for TSH and picomoles per milliliter for FT3 and FT4). The intra- and inter-assay coefficients of variation (CVs) were ≤3.7 and ≤8.6 for TSH, ≤6.4 and ≤5.5 for FT3, and ≤10.29 and ≤7.58% for FT4, respectively.

Insulin growth-factor 1 (IGF-1) was measured by radio-immunoassay assay (IGF1-RIACT, Cisbio Bioassays, Codolet, France) and concentrations are expressed in ng per milliliter. The intra-assay CV was ≤3.8 and the inter-assay CV ≤8.2%. Leptin was measured by enzyme-linked immuno-sorbent assay (BioVendor Human Leptin ELISA, Brno, Czech Republic) and concentrations are expressed in ng per milliliter. The intra-assay CV was ≤7.6 and the inter-assay CV ≤6.7%. Total adiponectin was measured by enzyme-linked immunosorbent assay (ALPCO, Salem, NH, USA) and concentrations are expressed in µg per milliliter. The intra-assay CV was ≤5.7 and the inter-assay CV ≤6.4%.

DHEA, TT, DHT, and E2 were assayed simultaneously by gas chromatography-mass spectrometry, as previously described (36). Briefly, deuterated steroid internal standards (CDN Isotopes, Inc., Point- Claire, Quebec, Canada) were added to all plasma samples, which were then extracted with 1-chlorobutane. The organic extracts were purified on conditioned high-purity silica LC-Si SPE columns (Varian, Les Ulis, France). All steroids were derivatized with pentafluorobenzoyl chloride (77253-1ml, Sigma-Aldrich, Steinheim, Germany). The final extracts were reconstituted in isooctane and then transferred to conical vials for injection into the GC system (GC-2010 Plus, Shimadzu, Japan), equipped with a 50% phenylmethylpolysiloxane VF-17MS capillary column (20 m x0.15 mm, internal diameter, 0.15 mm film thickness; Agilent Technologies, Les Ulis, France). A TQ8050 (Shimadzu, Japan) triple quadrupole mass spectrometer equipped with a chemical ionization source and operating in Q3 single-ion monitoring mode was used for detection. Concentrations were reported for DHEA in nmol per liter and for DHT, TT, and E2 in pmol per liter. The intra- and inter-assay CVs were ≤3.5 and ≤4.7 for DHEA, ≤2.2 and ≤2.1 for TT, ≤3.0 and ≤3.1 for DHT, and ≤3.5 and ≤4.1% for E2, respectively.

### Lipid Analysis

Total cord plasma cholesterol and triglyceride concentrations were determined enzymatically (DiaSys Diagnostic Systems GmbH; Holzheim, Germany) and the total lipid concentration calculated as previously described (37).

### Data and Statistical Analysis

All analyses were stratified by sex because of gender differences in hormone production and potential sexual dimorphism related to the effect of chlordecone. Continuous variables are described as means, medians, inter-quartile ranges, and percentiles. Mean ranks between unpaired groups were compared using the Mann Whitney test in descriptive bivariate analyses.

Cord-blood chlordecone concentrations were considered to be categorical (quartiles, based on their distribution in the population study) or continuous variables after log_10_ transformation. Chlordecone values below the LOD were imputed by a maximum likelihood estimation method (38).

Associations between *in utero* chlordecone exposure and hormones with ≥90% detectable values (TSH, FT3, FT4, DHEA, IGF-1, and adiponectin for both sexes, and leptin for girls) were analyzed by multiple linear regression, allowing calculation of the β regression coefficient and its 95% confidence interval (95%CI). Hormones were included in the model after the imputation of values < LOD (38) and a log_10_ transformation, as they were log-normally distributed. Hormones with 14.3 to 72.6% detectable values (DHT, TT, and E2 for both sexes, and leptin for boys; see **Table 3**) were dichotomized according to their LOD (< LOD *vs* ≥ LOD) and potential associations with *in utero* chlordecone exposure analyzed using multiple logistic regression models, allowing estimation of the odds ratio (OR) and its 95%CI.

The following maternal covariates were considered to be potential confounding factors: age at delivery (years), geographic origin (Caribbean *vs* European), body mass index (BMI, kg/m^2^), weight gain during pregnancy (insufficient, normal, excessive, or very excessive, according to the guidelines of the Institute of Medicine (39), education (< 12 years schooling *vs* ≥ 12 years schooling), smoking during pregnancy (never *vs* ever), and alcohol consumption during pregnancy (never *vs* ever). We also considered the following child covariates: exact age at examination (years), z-score height, z-score BMI, preterm birth (yes, no), small for gestational age (tenth percentile (40); yes, no), breastfeeding (yes, no), time of day when blood was drawn, and total cord-plasma lipid concentration (g/L). The characteristics of the samples were taken into account, as hemolysis was present in some (ranked by visual inspection as none or light, moderate, or strong) and could have affected the hormonal analysis. These covariates, including hemolysis, were included in all statistical models and then selected by applying a backward stepwise elimination procedure at *P* < 0.2. Missing values for each covariate were coded as an indicator variable to indicate missing. The Hosmer-Lemeshow goodness-of-fit test was used for the final logistic models.

We also implemented general additive models (GAM), including restricted cubic splines, to fit a potential non-linear association between *in utero* chlordecone exposure and hormone levels (41). Analyses were carried out using Stata 15.1 (College Station, Texas, USA) for linear and logistic regression modeling and SAS (SAS Institute Inc., North Carolina, USA) for GAM modeling. All tests were two-tailed, and *P* values <0.05 were considered statistically significant.

## Results

The mothers’ and children’s characteristics according to the sex of the child are presented in **Table 1**. Children included in the study had an average age of 7.7 years for boys and 7.6 years for girls. The geographical origin of the family was predominantly the Caribbean islands (~97%). The mothers’ mean age at delivery was 32 years and very few reported alcohol consumption (~3%) or smoking (~2%) during pregnancy. Thirteen percent of the children were born preterm.

**Table 1:** General characteristics of the study population according to sex.

VARIABLE	BOYS (N = 210)	GIRLS (N = 228)
**Mothers’ characteristics**		
Age at delivery (mean, min, max)	32.0 (16.2, 45.1)	32.0 (15.1, 44.8)
Geographic origin		
Caribbean Islands	204 (97.1)	219 (96.0)
Europe	6 (2.9)	9 (4.0)
BMI in early pregnancy (mean, min, max)	24.8 (17.1, 40.7)	25.7 (15.6, 50.0)
Weight gain during pregnancy (n, %)		
Insufficient	66 (32.2)	58 (25.8)
Normal	28 (13.7)	26 (11.5)
Excessive	53 (25.8)	78 (34.7)
Very excessive	58 (28.3)	63 (28.0)
Education level (n, %)		
< 12 years schooling	112 (53.3)	117 (51.3)
≥ 12 years schooling	98 (46.7)	111 (48.7)
Smoking during pregnancy (n, %)	6 (2.9)	7 (3.1)
Alcohol during pregnancy (n, %)	4 (2.0)	5 (2.3)
**Childs’ characteristics**		
Age (mean, min, max)	7.7 (7.1, 8.1)	7.6 (7.1, 8.2)
Z-score height (mean, min, max)	0.9 (-1.9, 3.8)	1.0 (-1.4, 3.6)
Z-score BMI (mean, min, max)	0.2 (-3.8, 3.7)	0.3 (-3.1, 2.9)
Prematurity (n, %)	29 (13.8)	28 (12.3)
Small for gestational age (n, %)	24 (11.4)	17 (7.5)
Breastfeeding (n, %)	183 (87.1)	192 (84.2)
Total cord blood lipids (mean, interquartile range) (g/L)	2.24 (1.82, 2.41)	2.29 (1.75, 2.55)

The distributions of organochlorine contaminants in cord blood and the blood of the children at seven years of age are presented in **Table 2**. The detection frequency and median concentration of chlordecone in cord blood was 79% and 0.25 µg/L for boys, and 76.4% and 0.21 µg/L for girls. At seven years of age, chlordecone was detected in 73.8% (median concentration = 0.06 µg/L) of the blood samples of boys and 71.1% (0.05 µg/L) of those of girls. DDE and PCB-153 were also detected in 79.5% (median concentration = 0.22 µg/L) and 56.7 (0.07 µg/L) of the cord blood samples of boys and 84.9% (0.31 µg/L) and 52.2% (0.06 µg/L) of those of girls, respectively. We observed no differences according to sex, regardless of the contaminants considered (**Table 2**). Spearman’s rank correlation values (rho) between cord chlordecone and DDE or PCB-153 levels were 0.16 (*P* = 0.002) and -0.01 (*P* = 0.83), respectively. Cord-blood and child chlordecone concentrations were poorly correlated (rho = 0.09, *P* = 0.14).

**Table 2:** Detection and concentrations (µg/l) of organochlorine contaminants in cord and child blood samples.

Organochlorine	N	Detection frequency (%)	Minimum	p5	p25	p50	p75	p95	Maximum	*P* ^a^
**Cord blood**										
Chlordecone										
Boys	124	79.0	< DL	< DL	0.08	0.25	0.41	1.46	12.5	0.46
Girls	161	76.4	< DL	< DL	0.07	0.21	0.37	1.44	29.8
DDE										
Boys	127	79.5	< DL	< DL	0.09	0.22	0.64	2.71	7.78	0.26
Girls	159	84.9	< DL	< DL	0.10	0.31	0.74	3.22	12.5
PCB-153										
Boys	127	56.7	< DL	< DL	< DL	0.07	0.15	0.46	1.31	0.69
Girls	159	52.2	< DL	< DL	< DL	0.06	0.14	0.57	1.75
**Child blood**										
Chlordecone										
Boys	210	73.8	< DL	< DL	< DL	0.06	0.11	0.37	7.01	0.37
Girls	225	71.1	< DL	< DL	< DL	0.05	0.11	0.30	2.15

DL, Detection limits. Cord chlordecone: 0.06 µg/L, cord DDE: 0.05 µg/L, cord PCB-153: 0.05 µg/L, child chlordecone: 0.02 µg/L.

**^a^** Mann-Whitney U test.

The distribution of hormone concentrations in the blood of the children at seven years of age is presented in **Table 3**. The detection frequency was 100% for TSH, FT4, FT3, IGF-1, and adiponectin for both sexes. The detection frequency for DHEA was 94.1% for boys and 99.1% for girls, whereas it was 91.7% for leptin for girls. The detection frequency was between 14.3 and 72.6% for the other hormones (DHT, TT, and E2 for both sexes, and leptin for boys). Girls showed significantly higher plasma levels of FT3, DHEA, TT, DHT, E2, IGF-1, and leptin than boys, whereas TSH, FT4, and adiponectin did not differ according to sex (**Table 3**). TSH, FT3 and FT4 levels were within normal range expected at the age of seven years (42). For the other hormones, available information is scarce and there are no well-established reference values in large populations of children at the age of seven years.

**Table 3:** Detection and concentrations of hormones in child blood samples according to sex.

Hormones	N	Detection frequency (%)	Minimum	p5	p25	p50	p75	p95	Maximum	*P* ^a^
TSH (mUI/L)										
Boys	210	100	0.59	0.99	1.49	2.10	2.88	4.19	7.15	0.22
Girls	226	100	0.35	0.85	1.44	2.00	2.63	4.28	6.63	
FT3 (pmol/L)										
Boys	210	100	2.90	4.90	5.50	6.20	6.90	8.10	12.7	0.05
Girls	228	100	3.70	4.70	5.60	6.40	7.10	9.40	12.8	
FT4 (pmol/L)										
Boys	210	100	13.2	14.9	15.9	16.9	18	20	21.6	0.70
Girls	228	100	13.3	14.6	15.6	16.85	18.2	20.3	26.9	
DHEA (nmol/L)										
Boys	203	94.1	< DL	< DL	1.63	2.93	5.03	8.94	19.9	0.01
Girls	219	99.1	< DL	1.00	2.23	3.44	5.69	10.1	29.0	
DHT (pmol/L)										
Boys	203	61.1	< DL	< DL	< DL	38.9	76.9	176.5	959.0	0.002
Girls	219	72.6	< DL	< DL	< DL	55.2	108.1	270.3	1112.3	
TT (pmol/L)										
Boys	203	36.0	< DL	< DL	< DL	< DL	112.2	261.7	438.9	0.02
Girls	219	46.1	< DL	< DL	< DL	< DL	140.1	264.4	712.2	
E2 (pmol/L)										
Boys	203	14.3	< DL	< DL	< DL	< DL	< DL	7.06	13.7	<0.0001
Girls	219	43.4	< DL	< DL	< DL	< DL	7.50	15.4	34.8	
IGF-1 (ng/mL)										
Boys	210	100	97	144	174	218.5	254	318	440	<0.0001
Girls	228	100	113	151	206.5	253.5	303.5	415	529	
Leptin (ng/mL)										
Boys	210	70.0	< DL	< DL	< DL	1.70	3.41	14.3	36.0	<0.0001
Girls	228	91.7	< DL	< DL	1.96	3.68	8.48	23.2	45.6	
Adiponectin (µg/mL)										
Boys	210	100	2.05	2.65	3.90	4.73	5.75	8.00	11.3	0.13
Girls	228	100	2.05	3.15	4.05	5.00	6.08	8.15	12.4	

DL, Detection limits. DHEA: 0.3 nmol/L, DHT: 28.3 pmol/L, testosterone 81.3 pmol/L, estradiol: 5.1 pmol/L, leptin: 1.0 ng/mL.

**^a^** Mann-Whitney U test.

The results of crude and adjusted linear or logistic regression analyses for *in utero* chlordecone exposure are presented in **Tables 4** to 6 for thyroid, metabolic, and sex-steroid hormones, respectively.

**Table 4:** Associations between *in utero* (cord blood) chlordecone exposure and thyroid hormone concentrations at seven years of age in children of the TIMOUN cohort.

Hormone	Sex (N)	Chlordecone (µg/L)	Unadjusted			Adjusted^a^		
			ß^b^	95% CI	*P*	ß^b^	95% CI	*P*
**TSH** ^b^ (mIU/L) (log_10_)	Boys (124)	<0.07	Ref.			Ref.		
		0.07-0.19	0.12	-0.12; 0.36	0.32	0.20	-0.04; 0.44	0.10
		0.20-0.40	0.01	-0.21; 0.23	0.92	0.05	-0.17; 0.26	0.68
		>0.40	0.02	-0.20; 0.24	0.85	0.10	-0.13; 0.33	0.38
		Log10	-0.01	-0.07; 0.04	0.71	-0.01	-0.05; 0.06	0.82
	Girls (159)	<0.07	Ref.			Ref.		
		0.07-0.19	0.10	-0.11; 0.32	0.34	0.10	-0.11; 0.32	0.51
		0.20-0.40	0.19	-0.02; 0.40	0.08	0.22	0.01; 0.44	0.04
		>0.40	0.05	-0.16; 0.27	0.63	0.08	-0.15; 0.30	0.50
		Log10	0.02	-0.04; 0.07	0.53	0.02	-0.03; 0.08	0.39
**FT3** ^b^ (pmol/mL) (log_10_)	Boys (124)	<0.07	Ref.			Ref.		
		0.07-0.19	-0.32	-0.93; 0.28	0.29	-0.22	-0.76; 0.32	0.43
		0.20-0.40	0.14	-0.42; 0.70	0.62	0.03	-0.46; 0.52	0.92
		>0.40	0.24	-0.33; 0.81	0.41	-0.06	-0.57; 0.44	0.80
		Log10	0.11	-0.03; 0.26	0.11	0.05	-0.08; 0.17	0.45
	Girls (161)	<0.07	Ref.			Ref.		
		0.07-0.19	0.30	-0.23; 0.83	0.27	0.32	-0.21; 0.85	0.24
		0.20-0.40	0.37	-0.15; 0.89	0.16	0.42	-0.11; 0.94	0.12
		>0.40	0.18	-0.36; 0.71	0.52	0.18	-0.36; 0.73	0.51
		Log10	0.07	-0.07; 0.20	0.33	0.08	-0.06; 0.22	0.27
**FT4** ^b^ (pmol/mL) (log_10_)	Boys (124)	<0.07	Ref.			Ref.		
		0.07-0.19	-0.32	-1.15; 0.50	0.44	-0.22	-1.08; 0.63	0.61
		0.20-0.40	-0.18	-0.95; 0.58	0.63	-0.25	-1.00; 0.51	0.52
		>0.40	-0.28	-1.06; 0.50	0.48	-0.35	-1.12; 0.41	0.36
		Log10	0.04	-0.15; 0.23	0.69	0.02	-0.17; 0.21	0.83
	Girls (161)	<0.07	Ref.			Ref.		
		0.07-0.19	-0.22	-1.06; 0.62	0.60	-0.13	-0.95; 0.69	0.76
		0.20-0.40	0.08	-0.75; 0.90	0.86	0.21	-0.61; 1.02	0.61
		>0.40	0.35	-0.50; 1.20	0.42	0.52	-0.32; 1.35	0.23
		Log10	0.11	-0.10; 0.32	0.30	0.17	-0.04; 0.38	0.12

a The covariates for which we adjusted: For TSH boys: mothers’ BMI in early pregnancy, alcohol during pregnancy; For TSH girls: alcohol during pregnancy, breastfeeding; For FT3 boys: geographic origin, mothers’ age at delivery, breastfeeding, alcohol during pregnancy; For FT3 girls: geographic origin, mothers age at delivery, alcohol during pregnancy; For FT4 boys: mothers’ age at delivery, breastfeeding, z-BMI; For FT4 girls: z-BMI.

b Beta coefficient of regression.

**Figure 1:**
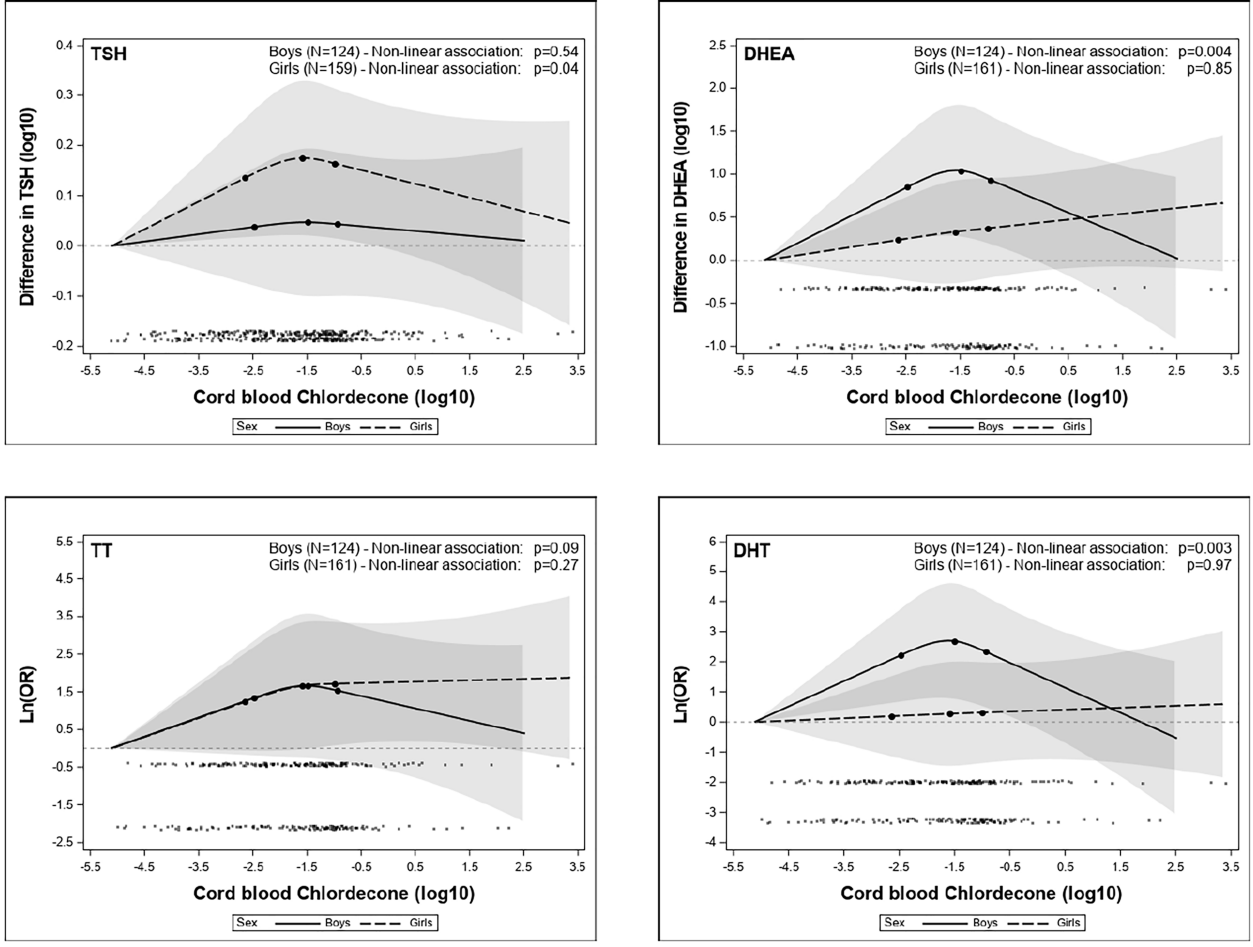
Exposure-response associations (adjusted generalized additive models, restricted cubic splines). Horizontal axis: log_10_ (cord blood chlordecone concentration, µg/L). Vertical axis: difference in log_10_ TSH (mIU/L), log_10_ DHEA (nmol/L), Ln (OR) TT (above *vs* below the detection limit), and Ln (OR) DHT (above *vs* below the detection limit) estimated for various levels of exposure compared to the minimal exposure observed. Black points at the bottom of the graph represent the observed values of chlordecone concentrations.

Multiple linear regression analysis showed TSH levels to be significantly higher in the third quartile of cord-blood chlordecone concentrations for girls than those in the lowest quartile (adjusted model, β = 0.22, 95%CI = 0.01-0.44; **Table 4**). Non-linear modelling using restricted cubic splines of the associations between cord-blood chlordecone concentrations (as continuous variables) and TSH levels in girls showed a significant non-linear trend (adjusted model, *P* = 0.04) (**Figure 1**). We obtained comparable results following additional adjustments for cord blood DDE or child blood chlordecone concentrations in the multivariable models (β = 0.18, 95%CI = -0.03-0.39, and β = 0.23, 95%CI = 0.01-0.45 for the third quartile relative to the lowest for cord-blood DDE and child blood chlordecone, respectively) (**Supplemental Table S1**). By contrast, we observed no association between *in utero* chlordecone exposure (as continuous values or categorized by quartiles) and FT4 or FT3 levels for either sex, regardless of the adjustment model (**Table 4** and **Supplemental Table S1**).

There were no significant associations between cord-blood chlordecone concentration (as continuous or categorized variable) and any metabolic hormones, whatever the adjustment model (**Table 5** and **Supplemental Table S2**).

**Table 5:** Associations between *in utero* (cord blood) chlordecone exposure and metabolic hormone concentrations at seven years of age in children of the TIMOUN cohort.

Hormone	Sex (N)	Chlordecone (µg/L)	Unadjusted			Adjusted ^a^		
			ß^b^ or OR^c^	95% CI	*P*	ß^b^ or OR^c^	95% CI	*P*
**IGF-1** ^b^ (ng/mL) (log_10_)	Boys (124)	<0.07	Ref.			Ref.		
		0.07-0.19	4.79	-24.6; 34.2	0.75	-7.20	-35.41; 21.01	0.61
		0.20-0.40	7.92	-19.2; 35.1	0.57	-1.96	-27.14; 23.23	0.88
		>0.40	1.65	-26.1; 29.4	0.91	-3.34	-28.79; 22.11	0.80
		Log10	1.63	-5.26; 8.51	0.64	-0.22	-6.57; 6.13	0.95
	Girls (161)	<0.07	Ref.			Ref.		
		0.07-0.19	-16.1	-49.1; 16.9	0.34	-15.6	-47.0; 15.8	0.33
		0.20-0.40	-13.1	-45.6; 19.3	0.42	-16.3	-47.6; 14.9	0.30
		>0.40	-9.4	-42.8; 24.1	0.58	-16.8	-49.1; 15.4	0.31
		Log10	0.35	-8.03; 8.73	0.93	-3.51	-11.6; 4.54	0.39
**Adiponectin** ^b^ (µg/mL) (log_10_)	Boys (124)	<0.07	Ref.			Ref.		
		0.07-0.19	0.62	-0.29; 1.53	0.18	0.79	-0.14; 1.72	0.10
		0.20-0.40	0.52	-0.32; 1.36	0.23	0.67	-0.17; 1.51	0.12
		>0.40	0.62	-0.24; 1.48	0.16	0.68	-0.17; 1.53	0.12
		Log10	0.15	-0.06; 0.36	0.17	0.18	-0.04; 0.39	0.11
	Girls (161)	<0.07	Ref.			Ref.		
		0.07-0.19	-0.32	-1.05; 0.41	0.39	-0.36	-1.08; 0.37	0.34
		0.20-0.40	-0.35	-1.07; 0.36	0.33	-0.32	-1.04; 0.39	0.37
		>0.40	0.06	-0.68; 0.80	0.87	0.06	-0.68; 0.79	0.88
		Log10	0.03	-0.15; 0.22	0.72	0.04	-0.15; 0.22	0.68
**Leptin** ^c^ (<LOD *vs* ≥ LOD)	Boys (124)	<0.07	Ref.			Ref.		
		0.07-0.19	1.75	0.45; 6.84	0.42	0.82	0.16; 4.30	0.81
		0.20-0.40	0.72	0.24; 2.19	0.57	0.36	0.09; 1.45	0.15
		>0.40	0.73	0.24; 2.26	0.59	0.40	0.10; 1.69	0.21
		Log10	0.86	0.65; 1.15	0.32	0.74	0.50; 1.08	0.12
**Leptin** ^b^ (ng/mL) (log_10_)	Girls (161)	<0.07	Ref.			Ref.		
		0.07-0.19	0.29	-0.16; 0.75	0.20	0.10	-0.15; 0.36	0.42
		0.20-0.40	0.30	-0.14; 0.75	0.18	0.02	-0.24; 0.27	0.91
		>0.40	0.29	-0.17; 0.75	0.22	0.004	-0.26; 0.26	0.98
		Log10	0.10	-0.02; 0.21	0.10	-0.003	-0.07; 0.06	0.94

a The covariates for which we adjusted: For IGF-1 boys: geographical origin, mothers’ age at delivery, z-BMI; For IGF-1 girls: geographical origin, mothers’ age at delivery, mothers’ BMI in early pregnancy, child z-score BMI; For Adiponectin boys: geographical origin, child z-score BMI; For Adiponectin girls: breastfeeding; For Leptin boys: mothers age at delivery, mothers education level, child z-score BMI; For Leptin girls: geographical origin, child z-score BMI.

b Beta coefficient of regression;

c Odds ratio.

In terms of sex-steroid hormones, we found significantly higher levels in the third quartile of cord blood chlordecone concentration than in the lowest quartile for DHEA (adjusted model, β = 0.54, 95%CI = 0.08-1.01, for boys; β = 0.36, 95%CI = 0.02-0.71, for girls), TT (adjusted model, OR = 3.22, 95%CI = 1.08-9.6, for boys; OR = 3.28, 95%CI = 1.32-8.17, for girls), and DHT (adjusted model, OR = 3.70, 95%CI = 1.29-10.6, for boys; OR = 3.20, 95%CI = 1.01-10.2, for girls) (**Table 6**). Supplementary adjustments for cord blood DDE or child blood chlordecone concentrations had a minimal impact on the estimates (**Supplemental Table S3**). By contrast, we observed no associations concerning E2, regardless of the adjustment model (**Table 6** and **Supplemental Table S3**). Non-linear modelling of the associations between cord blood chlordecone exposure as a continuous variable and sex-steroid hormone levels showed a significant non-linear trend for DHEA (*P* = 0.004) and DHT (*P* = 0.003) and a non-significant non-linear trend for TT (*P* = 0.09) for boys only in adjusted models (**Figure 1**).

**Table 6:** Associations between *in utero* (cord blood) chlordecone exposure and steroid hormone concentrations at seven years of age in children of the TIMOUN cohort.

Hormone	Sex(N)	Chlordecone (µg/L)	Unadjusted			Adjusted^a^		
			ß^b^ or OR^c^	95% CI	*P*	ß^b^ or OR^c^	95% CI	*P*
**DHEA** ^b^ (nmol/L) (log_10_)	Boys (124)	<0.07	Ref.			Ref.		
		0.07-0.19	0.20	-0.31; 0.72	0.44	0.26	-0.24; 0.75	0.30
		0.20-0.40	0.54	0.06; 1.03	0.03	0.54	0.08; 1.01	0.02
		>0.40	0.37	-0.12; 0.86	0.13	0.39	-0.08; 0.86	0.11
		Log10	0.05	-0.08; 0.17	0.47	0.04	-0.08; 0.16	0.51
	Girls (161)	<0.07	Ref.			Ref.		
		0.07-0.19	0.20	-0.16; 0.55	0.27	0.18	-0.17; 0.52	0.32
		0.20-0.40	0.41	0.06; 0.75	0.02	0.36	0.02; 0.71	0.04
		>0.40	0.29	-0.07; 0.64	0.11	0.22	-0.13; 0.57	0.21
		Log10	0.09	0.004; 0.18	0.04	0.08	-0.01; 0.16	0.09
**DHT** ^c^(<LOD *vs* ≥ LOD)	Boys (124)	<0.07	Ref.			Ref.		
		0.07-0.19	1.97	0.67; 5.78	0.22	1.81	0.61; 5.39	0.29
		0.20-0.40	3.69	1.29; 10.56	0.02	3.70	1.29; 10.6	0.02
		>0.40	1.16	0.43; 3.15	0.77	1.17	0.43; 3.19	0.76
		Log10	0.99	0.77; 1.28	0.96	0.99	0.77; 1.28	0.96
	Girls (161)	<0.07	Ref.			Ref.		
		0.07-0.19	0.80	0.31; 2.03	0.63	0.81	0.31; 2.17	0.68
		0.20-0.40	2.64	0.88; 7.90	0.08	3.20	1.01; 10.2	0.05
		>0.40	1.05	0.40; 2.79	0.92	1.13	0.41; 3.15	0.81
		Log10	1.07	0.83; 1.37	0.63	1.08	0.83; 1.41	0.57
**Testosterone** ^c^(<LOD *vs* ≥ LOD)	Boys (124)	<0.07	Ref.			Ref.		
		0.07-0.19	1.02	0.34; 3.04	0.97	1.07	0.32; 3.58	0.91
		0.20-0.40	2.29	0.84; 6.23	0.11	3.22	1.08; 9.58	0.04
		>0.40	0.94	0.33; 2.63	0.90	1.33	0.43; 4.07	0.62
		Log10	1.03	0.80; 1.33	0.82	1.12	0.85; 1.47	0.43
	Girls (161)	<0.07	Ref.			Ref.		
		0.07-0.19	1.67	0.68; 4.06	0.26	1.89	0.76; 4.72	0.17
		0.20-0.40	3.11	1.27; 7.62	0.01	3.28	1.32; 8.17	0.01
		>0.40	2.09	0.83; 5.09	0.12	1.96	0.78; 4.88	0.15
		Log10	1.27	1.01; 1.61	0.04	1.25	0.98; 1.59	0.07
**Estradiol** ^c^(<LOD *vs* ≥ LOD)	Boys (124)	<0.07	Ref.			Ref.		
		0.07-0.19	0.70	0.17; 2.81	0.61	0.65	0.16; 2.65	0.55
		0.20-0.40	1.10	0.33; 3.61	0.88	1.14	0.34; 3.81	0.83
		>0.40	1.03	0.30; 3.52	0.96	1.05	0.30; 3.61	0.94
		Log10	0.99	0.73; 1.36	0.96	1.00	0.73; 1.37	0.99
	Girls (161)	<0.07	Ref.			Ref.		
		0.07-0.19	0.67	0.27; 1.62	0.37	0.72	0.29; 1.77	0.47
		0.20-0.40	1.26	0.53; 3.00	0.60	1.36	0.56; 3.26	0.50
		>0.40	0.90	0.37; 2.19	0.82	0.88	0.36; 2.16	0.78
		Log10	1.05	0.84; 1.31	0.70	1.03	0.82; 1.30	0.78

a The covariates for which we adjusted: For DHEA boys: child z-score BMI; For DHEA girls: geographical origin; For DHT boys: breastfeeding; For DHT girls: mothers’ age at delivery, smoking during pregnancy, child z-score BMI; For Testosterone boys: geographical origin, alcohol during pregnancy, breastfeeding; For testosterone girls: geographical origin, mothers’ age at delivery; For Estradiol boys: mothers’ BMI in early pregnancy; For estradiol girls: mothers’ age at delivery.

b Beta coefficient of regression;

c Odds ratio.

## Discussion

In the TIMOUN Mother-Child Cohort Study, we examined the association between prenatal (*in utero*) chlordecone exposure and thyroid, metabolic, and sex-steroid hormone levels in children at seven years of age. This age is a critical period during the process of child development, as it occurs at the end of the period of adiposity rebound (between five and seven years) (43) and before the onset of puberty, corresponding to the pre-pubertal surge from the adrenal gland (adrenarche) (44).

Blood chlordecone levels were lower in children at seven years of age than those observed at birth. Cord blood levels resulted from the *in-utero* transfer from mothers to their babies during the period of inclusion of pregnant women (2004–2007). Chlordecone levels in children at seven years of age (2011–2015) are the sum of what remains of the body burden at birth, in addition to continuous postnatal exposure. Considering the half-life of chlordecone in blood (5 to 6 months) (2), most of the *in utero* contribution had disappeared at seven years of age. Since 2005, the health authorities have been implementing preventive measures to reduce chlordecone exposure of the population. This has resulted in a reduction in chlordecone exposure, as estimated by blood measurements in the general population (8).

We found a non-monotonic (inverted-U) association between *in utero* exposure to chlordecone, assessed by concentrations in cord blood, and TSH levels for girls and DHEA, TT, and DHT levels for both boys and girls. Only the third quartile of *in utero* exposure was associated with significantly increased hormone levels. Supplementary adjustments for *in utero* DDE or postnatal chlordecone exposure did not change the results. Complementary non-linear analysis (spline regression) confirmed a significant non-linear trend for TSH levels in girls and for DHEA and DHT levels in boys, as well as a statistically non-significant trend for TT levels in boys.

### Thyroid Hormones

In the TIMOUN Mother-Child Cohort Study, we previously reported that cord-blood chlordecone concentrations were monotonously associated with increased TSH levels at three months of age in boys only, without modification of FT4 or FT3 levels (33). At seven years, we no longer observed such a profile in boys. However, we observed this profile in girls but with a non-monotonic dose-response pattern. In our population study, 7 (4.4%) of the 159 girls showed a slight increase in TSH levels to between 4.5 and 6.6 mIU/L, with strictly normal free thyroid hormone levels. The clinical importance of such a slight increase in plasma TSH levels (below 10 mIU/L) and the precise upper limit of the normal range for plasma TSH levels is still debated (45, 46). Although we cannot predict the clinical significance of our observations, the natural history of slight TSH elevations in healthy children who were not being drug treated show spontaneous normalization of TSH values (47). The biological mechanisms by which chlordecone could affect the thyroid axis are still unknown. A series of *in vivo* studies reported thyroid disruption in embryo and adult rare minnows exposed to chlordecone (48). However, complementary *in vitro* and *in silico* experiments showed only weak potency for the interaction of chlordecone with thyroid-related proteins (including thyroid receptors α and β) and suggested that thyroid alterations could be attributed to its interactions with ERs (48). Thus, the observed thyroid alterations in fish may have resulted from the well-recognized estrogenic activity of chlordecone. Whether a similar biological mechanism by which chlordecone could affect the thyroid axis in humans, and possibly differentially according to sex, is yet to be established.

### Metabolic Hormones

We did not observe any association between *in utero* chlordecone exposure and metabolic hormone levels for either sex in our study population. To date, no experimental or toxicological studies have addressed the question of possible relationships between chlordecone exposure or its effects and metabolic hormones. During the follow-up of the TIMOUN Mother-Child Cohort at the age of seven years, we found no clear evidence supporting an adipogenic effect of *in utero* chlordecone exposure. Despite significantly higher adiposity in the third quartile of *in utero* chlordecone exposure (particularly in boys), we were unable to formally establish a significant non-linear trend (Costet N, Lafontaine A, Rouget F, Michineau L, Monfort C, Thomé JP, Kadhel P, Multigner L, Cordier S; unpublished data). Given the present results concerning metabolic hormones, it appears unlikely that these hormones mediate changes in adiposity, if any, in response to chlordecone exposure.

### Sex Steroid Hormones

We observed increased levels of DHEA, TT, and DHT in children at seven years of age, but not E2, for the same third quartile of cord-blood chlordecone exposure relative to the lowest quartile. These hormones are located in the successive classical pathways of sex steroid production that include androstenedione and androstenediol, two alternative intermediate steps between DHEA and TT that we did not measure in the present study (29). Such relationships suggest that the increase in TT and DHT levels may result from an initial increase in the level of the substrate DHEA, consistent with the law of mass action, although increased enzyme activity in these pathways (3-β-hydroxy steroid dehydrogenase, 17-β- hydroxy steroid dehydrogenase, 5-α reductase) cannot be excluded. However, E2 levels were not modified, regardless of the level of *in utero* chlordecone exposure. Chlordecone is a recognized inhibitor of aromatase, the enzyme that converts TT to E2 (26). Thus, we cannot exclude the possibility that chlordecone-mediated inhibition of aromatase prevents increased E2 levels, despite an excess of TT as substrate. Finally, the origin of the increased levels of DHEA may be from any step upstream of cholesterol involving liver cytochrome P450 enzymes. Interestingly, experimental studies in rodents have shown that chlordecone induces cytochromes P450 enzymes (49) and may impair cholesterol homeostasis and tissue distribution (27, 50).

### Non-Monotonic Dose-Response

The non-monotonic dose-response (NMDR) we observed between *in utero* chlordecone exposure and certain hormones (inverted U-shaped curve) is characterized by associations at intermediate exposure concentrations and no association at lower and higher exposure concentrations. Such a relationship is not entirely unexpected, as it is recognized that certain EDCs may exhibit such patterns in experimental studies (51, 52). NMDRs can arise from numerous molecular mechanisms, such as opposing effects induced by multiple receptors differing in their affinity, receptor desensitization, negative feedback with increasing dose, or dose-dependent modulation of metabolism (53). In the TIMOUN Mother-Child Cohort Study, we previously observed a similar NMDR between *in utero* chlordecone exposure and birth weight in overweight and obese mothers (13).

### Strengths and Limits

The main strengths of this study lie in its prospective design, its being a population-based cohort study, and the exposure and outcome measurements. Prenatal (*in utero*) chlordecone exposure was determined using cord-blood samples, providing a representative measure of fetal exposure during the entire pregnancy, because the half-life of chlordecone in blood is approximately six months (54) and mothers were continuously exposed *via* the dietary intake of contaminated foods (6). Co-exposure to other EDCs was also considered, such as that to DDE and PCB-153, as well as childhood chlordecone exposure. We simultaneously measured a large number of hormones using standardized methods and gas chromatography–mass spectrometry for sex-steroid hormones, a method considered to be the gold standard for steroid hormone assays (55). Nevertheless, associations we observed in the present study, including the U-shaped relationships, should be interpreted with caution and we cannot exclude that it may result from a chance finding resulting from residual confounding or multiple comparisons.

## Conclusion

This study shows that prenatal (*in utero*) exposure to chlordecone is associated with increased levels of TSH in girls and increased levels of DHEA, TT, and DHT in boys and girls in a non-monotonic dose-response relationship at seven years of age. Additional studies are necessary to explore the biological mechanisms involved in these associations and, in parallel, to identify whether such changes are predictive of a subsequent occurrence of disease.

## Data Availability Statement

The datasets presented in this article are not readily available because supporting data cannot be made openly available due to ethical concerns. The TIMOUN team can provide the data on request, subject to appropriate approvals. Requests to access the datasets should be directed to Gülen Ayhan, gulen.ayhan@inserm.fr.

## Ethics Statement

The studies involving human participants were reviewed and approved by Comité de Protection des Personnes Sud-Ouest et Outremer III (n° 2011-AOOSSI-40). Written informed consent to participate in this study was provided by the participants’ legal guardian/next of kin.

## Author Contributions

LM, PK, SC, and AO contributed to the conception and design of the study. FR and LMi contributed to the acquisition of data. LMi and CM organized the database and GA, NC, and LMu performed the statistical analyses. J-PT performed the chemical analysis and FG the hormonal analysis and both wrote the corresponding sections of the manuscript. GA and LMu wrote the first draft of the manuscript. All authors contributed to the revision of the manuscript, read, and approved the submitted version.

## Funding

This work was supported by grants from the General Health Directorate (DGS RMC11129NNA & R17142NN), and the Fondation de France (N° 69263).

## Conflict of Interest

The authors declare that the research was conducted in the absence of any commercial or financial relationships that could be construed as a potential conflict of interest.

## Publisher’s Note

All claims expressed in this article are solely those of the authors and do not necessarily represent those of their affiliated organizations, or those of the publisher, the editors and the reviewers. Any product that may be evaluated in this article, or claim that may be made by its manufacturer, is not guaranteed or endorsed by the publisher.
