# Supplementary material for: In Utero Chlordecone Exposure and Thyroid, Metabolic, and Sex-Steroid Hormones at the Age of Seven Years: A Study From the TIMOUN Mother-Child Cohort in Guadeloupe
Source: Front Endocrinol (Lausanne). 2021 Nov 22;12:771641. doi: 10.3389/fendo.2021.771641 (PMC8648082; doi:10.3389/fendo.2021.771641)
Supplement: Supplementary file 1 [file Table_1.docx]

Supplementary Material

**Table S1. Associations between *in utero* (cord blood) chlordecone exposure and thyroid hormone concentrations at seven years of age for children of the TIMOUN cohort. Supplementary adjustments.**

| **Hormone** | **Sex**  **(N)** | **Chlordecone (µg/L)** | **Supplementary adjustment for cord blood DDE ^a^** | | | **Supplementary adjustment for child chlordecone ^b^** | | |
| --- | --- | --- | --- | --- | --- | --- | --- | --- |
|  |  |  | ß ^c^ | 95%CI | *P* | ß ^c^ | 95%CI | *P* |
| **TSH** ^c^  (mIU/L) (log_10_) | Boys (124) | <0.07 | Ref. |  |  | Ref. |  |  |
|  |  | 0.07-0.19 | 0.17 | -0.08 ; 0.42 | 0.42 | 0.17 | -0.07; 0.40 | 0.17 |
|  |  | 0.20-0.40 | 0.05 | -0.18 ; 0.28 | 0.67 | 0.02 | -0.20 ; 0.23 | 0.87 |
|  |  | >0.40 | 0.10 | -0.14 ; 0.33 | 0.43 | 0.12 | -0.10 ; 0.35 | 0.28 |
|  |  | Log10 | 0.01 | -0.05 ; 0.07 | 0.79 | 0.01 | -0.04 ; 0.07 | 0.62 |
|  | Girls (159) | <0.07 | Ref. |  |  | Ref. |  |  |
|  |  | 0.07-0.19 | 0.01 | -0.21 ; 0.23 | 0.91 | 0.08 | -0.14 ; 0.30 | 0.47 |
|  |  | 0.20-0.40 | 0.18 | -0.03 ; 0.39 | 0.09 | 0.23 | 0.01 ; 0.45 | 0.04 |
|  |  | >0.40 | -0.01 | -0.23 ; 0.22 | 0.96 | 0.06 | -0.17 ; 0.29 | 0.60 |
|  |  | Log10 | 0.003 | -0.05 ; 0.06 | 0.92 | 0.02 | -0.04 ; 0.08 | 0.49 |
| **FT3** ^c^  (pmol/mL) (log_10_) | Boys (124) | <0.07 | Ref. |  |  | Ref. |  |  |
|  |  | 0.07-0.19 | -0.19 | -0.75 ; 0.38 | 0.52 | -0.18 | -0.73 ; 0.36 | 0.50 |
|  |  | 0.20-0.40 | 0.07 | -0.44 ; 0.59 | 0.78 | 0.01 | -0.48 ; 0.50 | 0.97 |
|  |  | >0.40 | -0.05 | -0.57 ; 0.47 | 0.85 | -0.07 | -0.57 ; 0.43 | 0.79 |
|  |  | Log10 | 0.05 | -0.08 ; 0.18 | 0.43 | 0.05 | -0.08 ; 0.17 | 0.49 |
|  | Girls (161) | <0.07 | Ref. |  |  | Ref. |  |  |
|  |  | 0.07-0.19 | 0.26 | -0.30 ; 0.83 | 0.36 | 0.31 | -0.24 ; 0.86 | 0.26 |
|  |  | 0.20-0.40 | 0.37 | -0.18 ; 0.92 | 0.19 | 0.41 | -0.14 ; 0.96 | 0.14 |
|  |  | >0.40 | 0.12 | -0.46 ; 0.70 | 0.69 | 0.18 | -0.39 ; 0.74 | 0.54 |
|  |  | Log10 | 0.06 | -0.09 ; 0.21 | 0.40 | 0.07 | -0.07 ; 0.22 | 0.31 |
| **FT4** ^c^  (pmol/mL) (log_10_) | Boys (124) | <0.07 | Ref. |  |  | Ref. |  |  |
|  |  | 0.07-0.19 | -0.20 | -1.10 ; 0.70 | 0.66 | -0.05 | -0.90 ; 0.79 | 0.90 |
|  |  | 0.20-0.40 | -0.16 | -0.96 ; 0.64 | 0.70 | -0.13 | -0.87 ; 0.61 | 0.73 |
|  |  | >0.40 | -0.31 | -1.11 ; 0.49 | 0.44 | -0.34 | -1.09 ; 0.40 | 0.36 |
|  |  | Log10 | 0.03 | -0.16 ; 0.23 | 0.74 | 0.04 | -0.15 ; 0.23 | 0.70 |
|  | Girls (161) | <0.07 | Ref. |  |  | Ref. |  |  |
|  |  | 0.07-0.19 | 0.05 | -0.82 ; 0.92 | 0.91 | -0.13 | -0.95 ; 0.69 | 0.75 |
|  |  | 0.20-0.40 | 0.23 | -0.61 ; 1.07 | 0.60 | 0.09 | -0.74 ; 0.91 | 0.84 |
|  |  | >0.40 | 0.45 | -0.42 ; 1.32 | 0.31 | 0.39 | -0.45 ; 1.23 | 0.36 |
|  |  | Log10 | 0.15 | -0.08 ; 0.37 | 0.19 | 0.14 | -0.07 ; 0.35 | 0.21 |

**^a^** The covariates for which we adjusted: For TSH boys: mothers’ BMI in early pregnancy, alcohol during pregnancy; For TSH girls: alcohol during pregnancy, breastfeeding; For FT3 boys: geographic origin, mothers age at delivery, breastfeeding, alcohol during pregnancy; For FT3 girls: geographic origin, mothers’ age at delivery, alcohol during pregnancy; For FT4 boys: mothers’ age at delivery, breastfeeding, z-BMI; For FT4 girls: z-BMI, including cord blood DDE.

**^b^** The covariates for which we adjusted: For TSH boys: mothers’ BMI in early pregnancy, alcohol during pregnancy; For TSH girls: alcohol during pregnancy, breastfeeding; For FT3 boys: geographic origin, mothers’ age at delivery, breastfeeding, alcohol during pregnancy; For FT3 girls: geographic origin, mothers’ age at delivery, alcohol during pregnancy; For FT4 boys: mothers’ age at delivery, breastfeeding, child z-BMI; For FT4 girls: child z-BMI, including child chlordecone.

^c^ Beta coefficient of regression

**Table S2. Associations between *in utero* (cord blood) chlordecone exposure and metabolic hormone concentrations at seven years of age for children of the TIMOUN cohort. Supplementary adjustments.**

| **Hormone** | **Sex**  **(N)** | **Chlordecone (µg/L)** | **Supplementary adjustment for cord blood DDE ^a^** | | | **Supplementary adjustment for child chlordecone ^b^** | | |
| --- | --- | --- | --- | --- | --- | --- | --- | --- |
|  |  |  | ß^c^ or OR^d^ | 95%CI | *P* | ß^c^ or OR^d^ | 95%CI | *P* |
| **IGF-1** ^c^  (ng/mL) (log_10_) | Boys (124) | <0.07 | Ref. |  |  | Ref. |  |  |
|  |  | 0.07-0.19 | -4.49 | -33.81 ; 24.83 | 0.76 | -4.91 | -33.68 ; 23.86 | 0.74 |
|  |  | 0.20-0.40 | -3.64 | -30.15 ; 22.88 | 0.79 | -0.11 | -25.85 ; 25.64 | 0.99 |
|  |  | >0.40 | -2.46 | -28.83 ; 23.91 | 0.85 | -3.82 | -29.63 ; 22.00 | 0.77 |
|  |  | Log10 | -0.25 | -6.74 ; 6.24 | 0.94 | -0.34 | -6.90 ; 6.21 | 0.92 |
|  | Girls (161) | <0.07 | Ref. |  |  | Ref. |  |  |
|  |  | 0.07-0.19 | -18.93 | -52.73 ; 14.86 | 0.27 | -17.82 | -49.58 ; 13.94 | 0.27 |
|  |  | 0.20-0.40 | -18.28 | -51.15 ; 14.59 | 0.27 | -16.25 | -48.30 ; 15.81 | 0.32 |
|  |  | >0.40 | -19.99 | -54.30 ; 14.32 | 0.25 | -23.21 | -55.83 ; 9.40 | 0.16 |
|  |  | Log10 | -4.45 | -13.14 ; 4.23 | 0.31 | -3.85 | -11.94 ; 4.23 | 0.35 |
| **Adiponectin** ^c^ (log_10_)  (µg/mL) | Boys (124) | <0.07 | Ref. |  |  | Ref. |  |  |
|  |  | 0.07-0.19 | 0.64 | -0.30 ; 1.58 | 0.18 | 0.79 | -0.15 ; 1.72 | 0.10 |
|  |  | 0.20-0.40 | 0.48 | -0.38 ; 1.34 | 0.27 | 0.61 | -0.24 ; 1.46 | 0.16 |
|  |  | >0.40 | 0.54 | -0.32 ; 1.39 | 0.22 | 0.62 | -0.23 ; 1.47 | 0.15 |
|  |  | Log10 | 0.15 | -0.06 ; 0.36 | 0.16 | 0.14 | -0.08 ; 0.36 | 0.20 |
|  | Girls (161) | <0.07 | Ref. |  |  | Ref. |  |  |
|  |  | 0.07-0.19 | -0.42 | -1.19 ; 0.36 | 0.29 | -0.35 | -1.10 ; 0.39 | 0.35 |
|  |  | 0.20-0.40 | -0.25 | -0.99 ; 0.49 | 0.51 | -0.31 | -1.04 ; 0.43 | 0.41 |
|  |  | >0.40 | 0.18 | -0.59 ; 0.95 | 0.64 | 0.03 | -0.73 ; 0.78 | 0.95 |
|  |  | Log10 | 0.08 | -0.12 ; 0.28 | 0.42 | 0.03 | -0.16 ; 0.22 | 0.75 |
| **Leptin** ^d^  (<LOD vs > LOD) | Boys (124) | <0.07 | Ref. |  |  | Ref. |  |  |
|  |  | 0.07-0.19 | 0.82 | 0.15 ; 4.56 | 0.82 | 0.83 | 0.15 ; 4.43 | 0.82 |
|  |  | 0.20-0.40 | 0.35 | 0.08 ; 1.54 | 0.17 | 0.42 | 0.10 ; 1.77 | 0.24 |
|  |  | >0.40 | 0.40 | 0.08 ; 1.89 | 0.25 | 0.43 | 0.10 ; 1.93 | 0.27 |
|  |  | Log10 | 0.73 | 0.49 ; 1.11 | 0.14 | 0.75 | 0.50 ; 1.13 | 0.17 |
| **Leptin** ^c^  (ng/mL) (log_10_) | Girls (161) | <0.07 | Ref. |  |  | Ref. |  |  |
|  |  | 0.07-0.19 | 0.11 | -0.17 ; 0.38 | 0.45 | 0.11 | -0.15 ; 0.36 | 0.40 |
|  |  | 0.20-0.40 | 0.03 | -0.23 ; 0.29 | 0.84 | 0.05 | -0.20 ; 0.31 | 0.68 |
|  |  | >0.40 | 0.03 | -0.25 ; 0.30 | 0.85 | 0.01 | -0.25 ; 0.27 | 0.95 |
|  |  | Log10 | 0.003 | -0.07 ; 0.07 | 0.93 | -0.02 | -0.07 ; 0.06 | 0.96 |

**^b^** The covariates for which we adjusted: For IGF-1 boys: grandparents geographical origin, mothers age at delivery, z-BMI; For IGF-1 girls: grandparents geographical origin, mothers age at delivery, mothers BMI in early pregnancy, child z-score BMI; For Adiponectin boys: geographical origin, child z-score BMI; For Adiponectin girls: breastfeeding; For Leptin boys: mothers’ age at delivery, mothers’ education level, child z-score BMI; For Leptin girls: geographical origin, child z-score BMI, including child chlordecone.

**^b^** The covariates for which we adjusted: For IGF-1 boys: geographical origin, mothers’ age at delivery, child z-BMI; For IGF-1 girls: geographical origin, mothers’ age at delivery, mothers’ BMI in early pregnancy, child z-score BMI; For Adiponectin boys: geographical origin, child z-score BMI; For Adiponectin girls: breastfeeding; For Leptin boys: mothers’ age at delivery, ‘ education level, child z-score BMI; For Leptin girls: geographical origin, child z-score BMI, including child chlordecone.

**^c^** Beta coefficient of regression ; ^d^ Odds ratio

**Table S3. Associations between *in utero* (cord blood) chlordecone exposure and steroid hormone concentrations at seven years of age for children of the TIMOUN cohort. Supplementary adjustments**

| **Hormone** | **Sex**  **(N)** | **Chlordecone (µg/L)** | **Supplementary adjustment for cord blood DDE ^a^** | | | **Supplementary adjustment for chlordecone at 7 yo  ^b^** | | |
| --- | --- | --- | --- | --- | --- | --- | --- | --- |
|  |  |  | ß^c^ or OR^d^ | 95%CI | *P* | ß^c^ or OR^d^ | 95%CI | *P* |
| **DHEA** ^c^  (nmol/L) (log_10_) | Boys (124) | <0.07 | Ref. |  |  | Ref. |  |  |
|  |  | 0.07-0.19 | 0.20 | -0.31 ; 0.71 | 0.44 | 0.31 | -0.18 ; 0.80 | 0.21 |
|  |  | 0.20-0.40 | 0.54 | 0.06 ; 1.03 | 0.03 | 0.61 | 0.14 ; 1.07 | 0.01 |
|  |  | >0.40 | 0.35 | -0.13 ; 0.84 | 0.15 | 0.36 | -0.11 ; 0.83 | 0.13 |
|  |  | Log10 | 0.03 | -0.09 ; 0.15 | 0.59 | 0.04 | -0.08 ; 0.16 | 0.48 |
|  | Girls (161) | <0.07 | Ref. |  |  | Ref. |  |  |
|  |  | 0.07-0.19 | 0.17 | -0.20 ; 0.53 | 0.37 | 0.13 | -0.22 ; 0.49 | 0.45 |
|  |  | 0.20-0.40 | 0.32 | -0.03 ; 0.68 | 0.07 | 0.39 | 0.04 ; 0.74 | 0.03 |
|  |  | >0.40 | 0.16 | -0.21 ; 0.53 | 0.39 | 0.20 | -0.15 ; 0.56 | 0.26 |
|  |  | Log10 | 0.06 | -0.03 ; 0.16 | 0.17 | 0.07 | -0.02 ; 0.16 | 0.10 |
| **DHT** ^d^  (<DL vs > DL) | Boys (124) | <0.07 | Ref. |  |  | Ref. |  |  |
|  |  | 0.07-0.19 | 1.81 | 0.58 ; 5.66 | 0.31 | 1.90 | 0.62 ; 5.85 | 0.26 |
|  |  | 0.20-0.40 | 4.11 | 1.34 ; 12.57 | 0.01 | 4.20 | 1.40 ; 12.59 | 0.01 |
|  |  | >0.40 | 1.09 | 0.39 ; 3.09 | 0.87 | 1.20 | 0.43 ; 3.31 | 0.73 |
|  |  | Log10 | 0.97 | 0.75 ; 1.26 | 0.84 | 1.01 | 0.78 ; 1.32 | 0.91 |
|  | Girls (161) | <0.07 | Ref. |  |  | Ref. |  |  |
|  |  | 0.07-0.19 | 0.68 | 0.24 ; 1.96 | 0.48 | 0.75 | 0.27 ; 2.12 | 0.59 |
|  |  | 0.20-0.40 | 2.92 | 0.87 ; 9.87 | 0.08 | 4.66 | 1.29 ; 16.83 | 0.02 |
|  |  | >0.40 | 0.95 | 0.32 ; 2.79 | 0.92 | 1.10 | 0.38 ; 3.21 | 0.86 |
|  |  | Log10 | 1.04 | 0.78 ; 1.37 | 0.81 | 1.09 | 0.83 ; 1.45 | 0.53 |
| **Testosterone** ^d^  (<DL vs > DL) | Boys (124) | <0.07 | Ref. |  |  | Ref. |  |  |
|  |  | 0.07-0.19 | 0.92 | 0.26 ; 3.32 | 0.90 | 1.10 | 0.32 ; 3.73 | 0.88 |
|  |  | 0.20-0.40 | 3.70 | 1.14 ; 11.98 | 0.03 | 3.42 | 1.12 ; 10.38 | 0.03 |
|  |  | >0.40 | 1.21 | 0.37 ; 3.95 | 0.75 | 1.30 | 0.42 ; 4.02 | 0.65 |
|  |  | Log10 | 1.10 | 0.83 ; 1.45 | 0.52 | 1.12 | 0.84 ; 1.48 | 0.44 |
|  | Girls (161) | <0.07 | Ref. |  |  | Ref. |  |  |
|  |  | 0.07-0.19 | 1.78 | 0.68 ; 4.66 | 0.24 | 2.13 | 0.83 ; 5.49 | 0.12 |
|  |  | 0.20-0.40 | 3.13 | 1.21 ; 8.05 | 0.02 | 3.96 | 1.51 ; 10.40 | 0.005 |
|  |  | >0.40 | 1.69 | 0.65 ; 4.37 | 0.28 | 2.07 | 0.80 ; 5.35 | 0.13 |
|  |  | Log10 | 1.22 | 0.95 ; 1.56 | 0.13 | 1.27 | 0.99 ; 1.63 | 0.06 |
| **Estradiol** ^d^  (<DL vs > DL) | Boys (124) | <0.07 | Ref. |  |  | Ref. |  |  |
|  |  | 0.07-0.19 | 0.70 | 0.17 ; 3.00 | 0.64 | 0.70 | 0.17 ; 2.90 | 0.62 |
|  |  | 0.20-0.40 | 1.37 | 0.40 ; 4.76 | 0.62 | 1.30 | 0.38 ; 4.45 | 0.68 |
|  |  | >0.40 | 1.17 | 0.33 ; 4.18 | 0.81 | 1.03 | 0.30 ; 3.60 | 0.96 |
|  |  | Log10 | 1.02 | 0.75 ; 1.39 | 0.90 | 0.99 | 0.72 ; 1.38 | 1.00 |
|  | Girls (161) | <0.07 | Ref. |  |  | Ref. |  |  |
|  |  | 0.07-0.19 | 0.79 | 0.31 ; 2.04 | 0.63 | 0.71 | 0.28 ; 1.79 | 0.46 |
|  |  | 0.20-0.40 | 1.58 | 0.64 ; 3.93 | 0.32 | 1.36 | 0.55 ; 3.39 | 0.51 |
|  |  | >0.40 | 0.99 | 0.39 ; 2.53 | 0.99 | 0.82 | 0.32 ; 2.09 | 0.68 |
|  |  | Log10 | 1.07 | 0.84 ; 1.37 | 0.57 | 1.02 | 0.81 ; 1.29 | 0.87 |

**^a^** The covariates for which we adjusted: For DHEA boys: child z-score BMI; For DHEA girls: geographical origin; For DHT boys: breastfeeding; For DHT girls: mothers’ age at delivery, smoking during pregnancy, child z-score BMI; For Testosterone boys: geographical origin, alcohol during pregnancy, breastfeeding; For testosterone girls: geographical origin, mothers’ age at delivery; For Estradiol boys: mothers’ BMI in early pregnancy; For estradiol girls: mothers’ age at delivery, including cod blood DDE.

**^b^** The covariates for which we adjusted: For DHEA boys: child z-score BMI; For DHEA girls: geographical origin; For DHT boys: breastfeeding; For DHT girls: mothers’ age at delivery, smoking during pregnancy, child z-score BMI; For Testosterone boys: geographical origin, alcohol during pregnancy, breastfeeding; For testosterone girls: geographical origin, mothers’ age at delivery; For Estradiol boys: mothers’ BMI in early pregnancy; For estradiol girls: mothers’ age at delivery, including child chlordecone.

**^c^** Beta coefficient of regression ; ^d^ Odds ratio
